# Reviewing the Literature on Professional Development for Higher Education Tutors in the Work-From-Home Era: Is it Time to Reconsider the Integration of Social Media?

**DOI:** 10.1007/s10639-021-10603-2

**Published:** 2021-07-02

**Authors:** Alejandro Acuyo

**Affiliations:** Lancaster University, Lancaster, England, UK

**Keywords:** Social Media, Higher Education, Networked Learning, Professional Learning Networks, Professional Development, Systematic Literature Review

## Abstract

Set in the context of higher education, this paper focuses on professional development-related challenges faced by teachers and specifically how these difficulties have been exacerbated by the recent Work-From-Home policy. The study investigates how the integration of social media into educators’ professional development plans can support tutors in this new status quo and prepare them for similar situations in the future. A systematic review of literature, based on a methodological instrument called PRISMA, identified 28 relevant articles for detailed analysis from an initial pool of 65. This revealed that social media-enabled professional development should be promoted across universities. The benefits include social media’s potential to provide tutors with a bespoke experience, that is specific to their evolving needs. Also notable, is social media’s potential to clear physical and temporal hurdles, resulting in a significantly more extensive professional learning network. This leads to faculty who are likely to reap the benefits of networked learning, by using social media as the infrastructure through which to establish a higher volume of more geographically dispersed connections to like-minded individuals. Institutions will need to tackle hurdles, namely faculty resistance to using this novel platform, as well as the anxiety of participating in open online spaces. This should be addressed by pacing the integration of social media-enabled professional development and by blending it with the more established practice of face-to-face workshops. This hybrid model will provide time and support for sceptical teachers to make the transition towards the integration of social media into their PD.

## Introduction

The purpose of this research is to investigate the potential value of integrating social media into the professional development (PD) plans of higher education (HE) teachers who have recently shifted online. By exploring the opportunities and challenges, recommendations will be made as to the role of social media-enabled learning in HE today.

### Research Focus

Given the recent Work-From-Home (WFH) mandate that has been enforced globally in response to the Covid-19 pandemic (Crawford et al., 2020), educators are under increasing pressure to adapt to this new status quo. More specifically, there is anecdotal evidence to suggest that formal PD opportunities are waning as a result of physical isolation from colleagues as well as the tangible work environment. PD has become deprioritized as teachers scramble to perform their role in the new online arena. This increased workload has particularly impacted tutors’ abilities to attend formal, stand-alone PD events (Oddone et al., 2019).

In response, now seems an opportune moment to review literature around the integration of social media into PD, in order to identify any possible gaps. Upon finding these gaps, this study seeks to make relevant recommendations as to how they can be filled. Moreover, an exploration of material related to the use of social technology, underpinned by the related concepts of professional learning networks (PLNs) and networked learning (NL), will provide HE stakeholders with a better understanding of plausible alternatives to traditional, face-to-face (F2F) PD. Unlike the disconnected gatherings associated with this long-established practice (Oddone et al., 2019), social media-enabled PD is undertaken online, gradually and without temporal or physical restrictions (Weeks, 2012 as cited in Rheingold, 2012). These features enable it to fill the PD gap that the WFH policy has created.

### Defining Key Concepts

Given the wide scope of the research area, the definition of ‘social media’ will be crucial. This study is not constrained to a single social networking site (SNS), but rather on social media’s overall potential as an enabler of PD. This is achieved through the adoption of Carr and Haye’s (2015) broader definition of social media:

“internet-based channels that allow users to opportunistically interact and selectively self-present, either in real-time or asynchronously, with both broad and narrow audiences who derive value from user-generated content and the perception of interaction with others” (p.50).

To unpack this, the ‘channels’ include examples of SNS platforms such as LinkedIn, but also cover micro-blogging tools such as Twitter and media-sharing sites such as YouTube (Donelan, 2016). The ‘interactions’ refer to HE educators’ communication with close colleagues (‘narrow audiences’) as well as geographically distant professionals (‘broad audiences’). Finally, the ‘value’ refers to PD opportunities generated from the shared ideas and material (‘user-generated content’) in an explicitly public arena (‘perception of interaction with others’).

Having established the definition of social media, it is now important to clarify what is meant by PD. This study argues away from the traditional, one-time training events view of PD (Guskey, 2002) and towards lifelong learning that is enabled through informal and gradual engagement with like-minded professionals (Postholm, 2012).

### Research Questions (RQs)

Overarching Question: To what extent, if at all, should social media be integrated into the PD of HE tutors?

RQ1: What are the potential PD-related benefits and opportunities created by social media?

RQ2: What are the potential PD-related challenges and shortcomings created by social media?

### Ontological and Epistemological Perspectives

The author of this paper views reality as a socially constructed concept, as opposed to a fixed, pre-existing entity that is awaiting discovery (Scotland, 2012). The RQs have been aligned to reflect this, in that they seek out subjective opinions of individuals by focusing on ‘opportunities’ and ‘challenges,’ which are both open to independent interpretation. Moreover, the focus of this study on tutors’ individually perceived value of social media-enabled PD, along with the synthesis of a range of literature where authors do not always agree, aligns the investigation with an ontologically constructionist assumption alongside a matching epistemologically interpretivist one. The former refers to the author’s stance that reality is subjective (Kivunja & Kuyini, 2017) and the latter underlines the position that facts cannot be separated from personal social values (Hodgson et al., 2012).

## Methodology and Approach

A systematic literature review (SLR) approach has been chosen for its potential to eliminate bias as a robust method (Boell & Cecez-Kecmanovic, 2015). The level of rigour involved is likely to make this paper more replicable (Levy & Ellis, 2006) and hence reliable. Details such as the inclusion criteria and the number of relevant results produced at each stage of the review are examples of how the study could be straightforwardly cross-checked by readers.

**Fig. 1:**
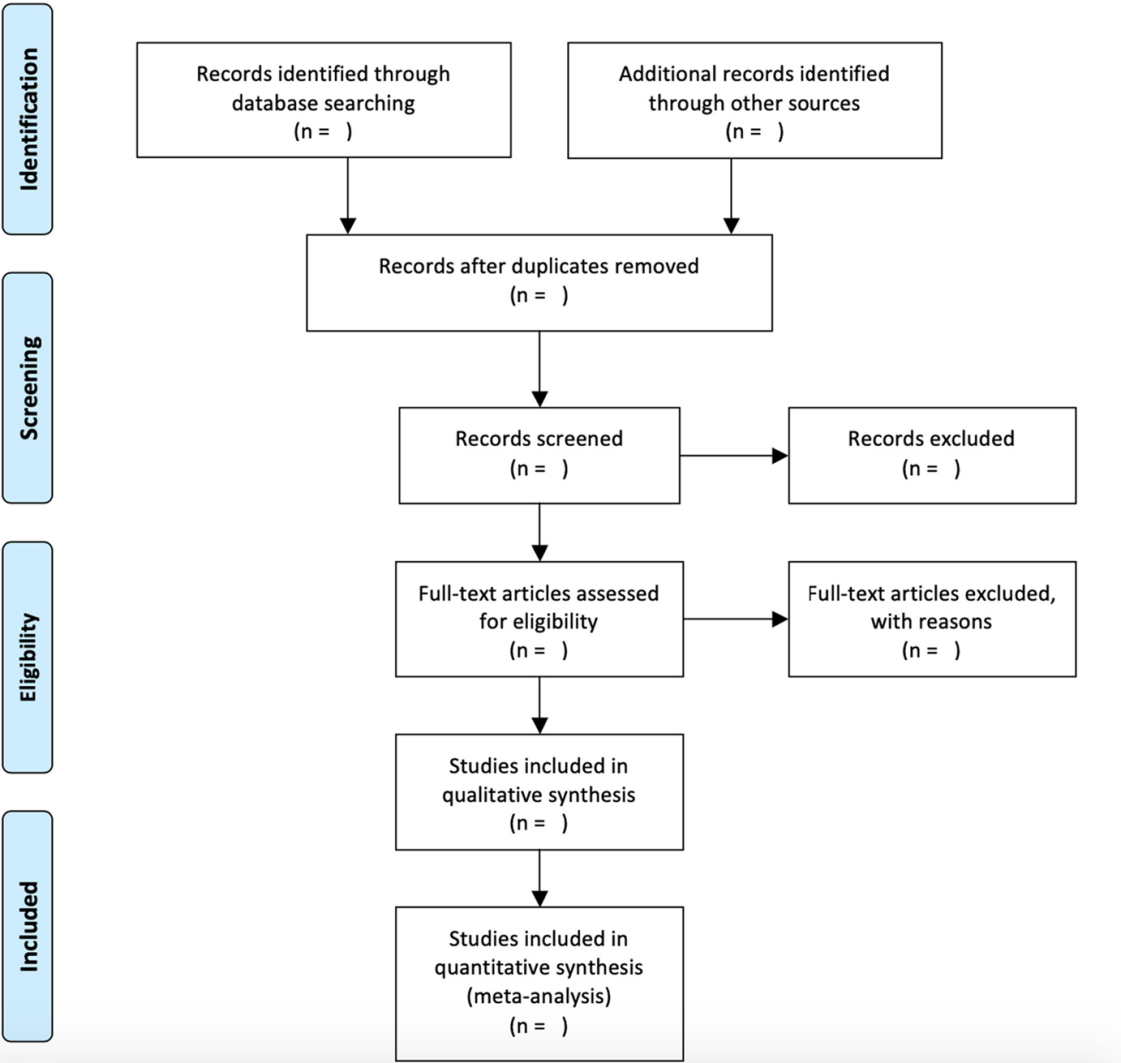
PRISMA Flow-Chart.

As a further safeguard against bias, this review was guided by the methodological instrument of ‘Preferred Reporting Items for Systematic Reviews and Meta-Analyses’ (PRISMA). This set of principles promotes transparent reporting in SLRs (Tricco et al., 2018) and is underpinned by a comprehensive checklist (Appendix 1) alongside a user-friendly flow-chart (Fig. 1) that highlights the main stages. This instrument was used as a guide, rather than as a script for the SLR. For instance, point 15 concerning the ‘risk of bias’ and point 25 regarding ‘funding’ were not applicable to this study, since the author has no conflict of interest or received funding for this study. Point 9 regarding the ‘study selection’ however, can be seen in the subsequent selection criteria section. https://doi.org/10.1371/journal.pmed1000097

### Selection Criteria

Having justified the methodology, this section details the conditions that sources must meet in order to be included in the final list of literature for analysis:

1. They must be English academic sources available in: Lancaster University’s One Search, Educational Resources Information Centre (ERIC) or Google Scholar. This is to balance databases with a large pool of sources (e.g. Google Scholar) versus those that are gate-kept for quality control filtering (e.g. ERIC).

2. They must relate to specific keyword combinations: Social Media; Higher Education; Networked Learning; Professional Learning Networks; Professional Development. This is because these keywords are closely aligned to the RQs.

3. They must be from within the 2010 to 2020 date range, except for literature used to support dated concepts (e.g. learning theories or epistemology). This is because social media has largely come into the public sphere over the past decade.

### Data Analysis

Table 1 details the four stages of the analysis in accordance with the PRISMA guidelines (Fig. 1). This includes the number of articles identified at each stepping-stone alongside the inclusion or exclusion rationale.

**Table 1:** Analysis Stages

Stage	Total Number of Sources	Explanation
1. Identification	628,077	The total number of results that appeared in the initial search using relevant keyword combinations from the three chosen databases before duplicates were removed a) Social Media AND Professional Development AND Higher Education: One Search: 398,729 Google Scholar: 52,700 ERIC: 135 b) Social Media AND Professional Development AND Higher Education AND Networked Learning: One Search: 10,945 Google Scholar: 19,100 ERIC: 4 c) Social Media AND Professional Development AND Higher Education AND Professional Learning Networks: One Search: 101,565 Google Scholar: 18,700 ERIC: 36 d) Social Media AND Professional Development AND Higher Education AND Networked Learning AND Professional Learning Networks: One Search: 7,259 Google Scholar: 18,900 ERIC: 4
2. Screening	65	The total number of sources that met the inclusion criteria after abstracts were examined from the first five pages (each page contained approximately 10 results) of the search results from each of the three databases (e.g. an article that focused on cyber security instead of PD was excluded)
3. Eligibility	49	The number of sources that were deemed to have met the inclusion criteria after the full texts were skim-read (e.g. an article that focused on K-12 schools instead of HE was excluded)
4. Included	28	The final number of articles that were finally read carefully and deemed to have met the inclusion criteria (e.g. an article that focused on classroom use of social media instead of PD was excluded)

A ‘screening document’ (Appendix 2) was used to search for duplicates in stages 2 and 3. The articles from section 4 were then synthesized using a literature matrix with numbered and annotated sources (Appendix 3). Lastly, a ‘synthesis notes’ document (Appendix 4) was used in conjunction with this matrix, in order to extrapolate ideas from the literature and link them to each individual source number. In some cases, secondary sources that were found within primary sources from the screening phase were located, read and added in addition to the final pool of included sources.

## Review of Literature

This SLR begins with an overview of the main themes from the target literature, before targeting both RQs individually. Lastly, the gaps are identified.

### Main Existing Conclusions

There is consensus among authors that PD, in its current form of F2F and often single-themed events, does not meet the needs of HE educators (Cook et al., 2017; Copper & Semich, 2014). Specific criticism includes the ‘one size fits all’ lack of tailoring to individuals’ contexts (Sindelar et al., 2010 as cited in Cook et al., 2017), time and temporal constraints and lack of continuity. These disadvantages of physical PD events have been exacerbated by the recent WFH policy. Prensky (2008 as cited in Copper & Semich, 2014) proposes that the onus is now on teachers to upgrade their technological competence, in order to adapt to survive in this newfound paradigm shift into the virtual workspace. One way in which this can be done is through social media.

Multiple authors argue that social media’s growing popularity has made it a ubiquitous and inescapable part of our lives (Zhu et al, 2018; Lieberman & Mace, 2010). This can be seen at institutional level through universities’ social media recruitment campaigns (Donelan, 2016), at student level where SNSs are being pedagogically integrated into classrooms and finally at faculty level as a PD platform. Authors have somewhat overlooked the digital divide (Hill & Lawton, 2018) that is attributed to how generational, household income and even geographical factors affect online access, when boasting social media’s prominence. Despite this, it is difficult to downplay the overt impact of social networks on HE over the past decade.

A significant amount of literature takes a broad comparative scope of multiple platforms (Cook, 2017; Schieffer, 2016), as opposed to focusing exclusively on one. These tools range from mainstream SNSs such as Facebook, to more specific academic-themed services such as ResearchGate. Despite the availability of some studies that focus exclusively on social media-enabled PD through YouTube’s media-sharing platform (Copper & Semich, 2014) or Linkedin’s professional networking SNS (Yap & Wang, 2015) for instance, the majority of authors centre on the micro-blogging platform known as Twitter (Greenhalgh & Koehler, 2016; O’Keeffe, 2016; Tucker, 2018). This is connected to this SNS’s potential to bridge local teaching communities with like-minded professionals dispersed across the globe (Forte et al., 2012). However, given that this could be argued in favour of other PD-enabling social media platforms, it is not clear from the existing literature what it is specifically that makes Twitter stand out. One proposal is that Twitter originally became a popular space for academics to share ideas on scholarship (Costa, 2013 as cited in O’Keeffe, 2016) and that this evolved into its use for more general teacher PD.

### RQ1: Benefits and Opportunities

Having considered general points of consensus among authors, this section will now target the first RQ by addressing the benefits.

The allure of social media-enabled PD can, at first glance, be attributed to its removal of time and temporal constraints (Cook et al., 2017; Trust et al., 2016). This means that a teacher seeking to engage in PD is no longer confined to the specific time parameters of a live session, since they can ‘reply,’ ‘share’ or otherwise engage with content asynchronously at a time that suits them (Hollins-Alexander, 2016). In a similar vein, social media’s potential to reach users worldwide means that teachers are no longer limited to the relatively small communities within their physical reach. Instead, PD-seeking teachers can now use social media to cross-pollinate their ideas (Forte et al., 2012) with a higher number of like-minded SNS users (Bedford, 2019; Bieke & Maarten, 2012) that are more geographically dispersed. This addresses the obstacles of isolation and time-pressure that the WFH directive has accelerated.

These benefits can partly be explained through NL, which is defined as the establishment and maintenance of connections between individual learners, group communities and educational resources through technology (Goodyear et al., 2004). Jones (2012) emphasizes the importance of these connections and how dependent NL’s success is on the ability of an individual to maintain these bridges. One can therefore conclude that unlocking the potential benefit of social media-enabled PD is not solely contingent on how teachers use social networks, but also on the technological features of the SNSs to facilitate the links with other professionals and resources. A platform that lacks key interactive functions, such as Twitter’s user-friendly ‘retweet’ feature than enables users to forward a succinct message to thousands of others or Facebook’s choice of reactions that enable users to express their emotions towards a post, is unlikely to prevail. This means that social media’s potential as a PD tool requires both widespread ‘buy in,’ ensuring a large enough pool of users and resources (Anders, 2018; Dabbagh & Kitsantas, 2012), alongside the infrastructure to support this scale of use. The lightning speed at which social media has advanced and risen in popularity across the last decade (O’Keeffe, 2016; Zhu et al., 2018) would give teachers reason to be optimistic.

This removal of time and physical barriers leads on to another key benefit of social media-enabled PD: its tailoring to each individual (Anders, 2018; Trust et al, 2016). Given that a teacher’s needs, objectives and motivation for PD are likely to change across their careers (Trust et al., 2017), they require a malleable platform that can support the fluidity of these shifts. Social media’s role as a centralised system through which teachers can effortlessly ‘un-follow’ professionals whose ideas no longer align with their own changing PD goals, and just as easily establish new connections that are more closely calibrated with their evolved aims (Anders, 2018), make it an appealing platform for today’s increasingly pressurized educators (Bedford, 2019; Donelan, 2016). That is to say, educators no longer need to search for a PD event that best aligns with their own needs, since social media offers PD that is already tailored to each tutor’s particular situation (O’Keeffe, 2018). A teacher who is specifically interested in materials design for medical students for instance, no longer needs to attend generic F2F sessions on materials design. Instead, they can seek out professionals with the same needs using an SNS, such as Twitter, to engage in two-way interaction with them and their materials.

This advantage of adopting a tailor-made approach to PD can be explored through a PLNs lens. Described as the unique web of personal contacts and spaces that individuals surround themselves with for PD purposes (Trust et al., 2017), PLNs place bespoke practice to meet the individual teacher’s needs at the forefront. The objective for PLNs’ participants is to build a series of micro-relationships with multiple users, in order to exchange ideas that help each other steadily grow and innovate professionally (Bedford, 2019; Atkins et al., 2016). In parallel with NL, PLNs are all about creating a bespoke network that meets the needs of each individual (Dabbagh & Kitsantas, 2012; Tucker, 2018). An English language educator may benefit from engaging with another English language instructor for the purposes of content-knowledge sharing but may also be interested in interacting with a teacher from a different subject, with the aim of sharing classroom-management strategies that would be applicable across disciplines for instance. Originally designed for physical spaces such as staff break rooms and conference halls, PLNs’ shift to virtual spaces has removed brick and mortar confinements (Kukulska-Hulme, 2012). This means that the participant pool that each social media-user has access to through virtual PLNs has been significantly amplified both in terms of volume as well as geographical dispersion (Lewis & Ewing, 2016).

Lastly, aside from the overt PD goal of gaining new knowledge through social and informal learning (O’Keeffe, 2016; Tucker, 2018) via naturally occurring user-to-user interaction, social media enabled-PD can provide more subtle benefits. These include a sense of belonging to a community (Dabbagh & Kitsantas, 2012; Meishar-Tal & Pieterse, 2017), professional support (Anders, 2018) and a platform with which to disseminate one's own ideas (Weller et al., 2014). The first benefit refers to the sensation of connectedness and professional identity that SNSs can facilitate, to create a sense of camaraderie among like-minded professionals who all face the same joys and challenges of teaching together as a single collective unit. Secondly, the guidance and reassurance that is on offer from users who are physically far away, but close in terms of profession and mindset, is likely to raise teachers’ morale. Finally, the two-way interaction supported by SNSs encourages teachers to actively engage (Bedford, 2019; Tucker, 2018) via ‘comments’ or ‘replies.’ This contrasts with the more passive trainer-led events that are associated with traditional workshop PD (Bedford, 2019) that is not supported by social media and often leaves participants voiceless (McConnell et al., 2013) as a result of its one-way communication model.

### RQ2: Challenges and Shortcomings

Having addressed the opportunities of social media-enabled PD, this section will now tackle the second RQ by considering the drawbacks.

A common barrier that arises upon first glance of the literature is the digital competence required to use social media confidently (Donelan, 2016; Zhu et al., 2018) to achieve one’s PD aims. The inability to effortlessly perform functions such as sharing content or sending a private message to engage directly with another participant restricts users to an experience of limited interaction (Cater et al., 2013; O’Keeffe, 2018) that does not take advantage of social media’s potential to engage with multiple users in a short space of time. This would prevent participants from reaping the benefits of NL, since it is dependent on a teacher’s ability not only to establish connections, but to maintain these through regular interaction (Goodyear et al., 2004). These functional barriers (Hu et al., 2011) encountered by teachers who are not able to confidently navigate platforms such as Twitter would therefore require investment in training, mentoring (Donelan, 2016; Zhu et al., 2018) and promotion of the platform, for the potential benefits of social media-enabled PD to be realised. While some educators who use social media for recreational purposes may not require this training, institutions would need a PD policy that is inclusive of all employees. This includes teachers who may be using social media for the first time. Even proficient users may require content suggestions to help them decide who to follow on Twitter in order to best meet their PD aims for instance. The financial and time contribution that this requires, means that buy-in from teachers and institutions alike would be a requisite.

A less overt hurdle that educators face is negative perceptions associated with social media (Hu et al., 2011), which could in turn lead to resistance in its adoption for PD purposes. This lack of faculty ‘buy-in’ (Cater et al., 2013) may be caused by connotations of social media as mere recreation tools for the younger generation, rather than professional platforms adopted by career-advancing educators. This lack of credibility is partly caused by the informal and unstructured nature of social media-enabled PD (Dron & Anderson, 2014), which means that participants are left to police the relevance and quality of their own content (Ranieri, 2019). This contrasts with the structure and comparative legitimacy that is offered by traditional, F2F workshops that are planned in advance and quality-guaranteed by the trainer(s). The reluctance to adopt social media as a PD platform can therefore be connected to the self-consciousness of some educators, who are concerned about its effect on their personal brand (Donelan, 2016).

Upon closer inspection of the literature, it appears that negative perceptions of social media are also caused by the sense of vulnerability and risk that is attributed to it, which can consequently inhibit active user participation (O’Keeffe, 2018). This refers to the danger that some users associate with sharing content and opinion with the intimidatingly high number of viewers that SNSs can support (Stewart, 2015), some of whom could publicly respond in disapproval or even resort to hate-speech (Ranieri, 2019). SNSs therefore risk empowering more self-assured users to dominate interactions (Robson, 2015), whilst diffident members are left ‘lurking’ on the peripheries (Bedford, 2019; Lorenzo & Aarcon-del-Amo, 2012). By adopting a social media platform for PD, users thus risk paying the price of public discomfiture, in order to reap the benefit of access to this wider network of professionals. Users who are unprepared to take this risk are unlikely to benefit from a PLN that is bespoke to their own specific needs (Trust el al., 2017), since they will be relegated to attending traditional PD events that target a general audience impersonally. This risk can be mitigated through carefully designed features within the SNS platforms themselves that cater to more timid participants (Bedford, 2019). An example of this is the inconspicuous ‘like’ button, which allows users to interact with content without the comparatively riskier ‘comment’ option, that is more likely to attract attention.

### Gaps

Assessing the target literature on the whole, there are a lack of in-depth case studies showcasing what a typical day in the life of a social media-enabled PD practitioner entails (Manca & Ranieri, 2017). It is true that there are exceptions. Notably De Laat’s (2012) practice-driven research programme examines individuals’ perceptions of NL through social media, via concept maps that specify participants’ specific connections to other members of the network at an institution in the Netherlands (Appendix 5). Even this, however, is limited to representing physical and bounded online space-based PLNs, via membership-restricted LMSs for instance within a single institution, as opposed to mapping the more complex networks that are formed via virtual PLNs in open online spaces via SNSs (Cronin, 2014). Another rare example of an investigation that homes in on individual case studies is Bedford’s (2019) study of how faculty develop virtual PLNs for PD at an American university. While this provides snippets of participants’ individual experiences via direct quotations that offers readers an overall gist of results, it does not consistently follow any one participant’s journey in detail.

Despite exceptions, most of the targeted literature describes social media-enabled PD in a more abstract and hypothetical manner (Cook et al., 2017; Trust el al., 2017; Cater et al., 2013), with comparatively little focus on in-depth individual experiences (Tour, 2017). For instance, Cook et al. (2017), detail a criterion that helps educators decide on a certain SNS according to their specific PD goal (Appendix 6), but do not follow any individual educator’s journey microscopically from beginning to end, in order to gain a deeper understanding of what is often referred to as the ‘invisible’ process (De Laat, 2012) of NL. This refers to the informal learning process via short, unplanned interactions with others as knowledge develops. In future, more phenomenographic studies focusing exclusively on fewer individuals’ detailed experiences, rather than studying larger samples in less detail, would help to fill this gap. This would help researchers to better understand the intricacies of social media-enabled participation in practice (De Laat, 2012), rather than treating it as an abstract, far-away concept.

Another notable gap is the blending of multiple approaches to PD. Authors often set ultimatums such as traditional F2F PD, in the form of isolated events, versus a more modern and continual lifelong learning approach online (Copper & Semich, 2014; Greenhalgh & Koehler, 2016; Kukulska-Hulme, 2012); yet little is written on combining more than one approach. Future studies should focus, for instance, on how the disadvantages of unstructured and unregulated learning attributed to social media-enabled PD (De Laat, 2012; Dron & Anderson, 2014) could be minimized by supplementing it with the legitimacy of traditional F2F training events (Ranieri, 2019). Understanding more about how the two approaches complement each other, rather than the over-simplification of praising one approach while demonizing the other, would help researchers find a compromise to keep more stakeholders satisfied.

Similar binary contrasts include the adoption of social media for close-knit network collaboration, promoted through Lave and Wenger’s (1991) Communities of Practice (CoPs) concept, whereby individuals learn by cooperating on a common project together, versus NL’s concept of networked individualism (Jones, 2012) whereby a participant learns by dipping in and out of a wider range of more loosely-tied networks (Luo et al., 2020). Again, there is a hole in the literature in terms of how the two concepts can be combined to minimize barriers and offer users the best of both worlds. For instance, a novice educator seeking basic knowledge on a single topic may be content to participate peripherally (Lave & Wenger, 1991) by working alongside a small PLN of more confident colleagues on a collaborative task. An example might be a new teacher who wants to learn about material design, so they agree to be mentored by colleagues who are already creating materials on YouTube. In contrast, a teacher from the same institution but working on an ambitious multi-disciplinary piece of action-research, may benefit more from a NL approach (Jones, 2012) to social media-enabled PD, by engaging in a range of micro-interactions with a wider range of professionals from a series of looser-knit networks. This could be someone writing a comparative paper on motivation among different HE professionals; they would need small-dose engagement with different discipline circles via a platform such as Twitter.

## Conclusions and Recommendations

This study has temporal limitations, as well as the fact that it relies on secondary data. Despite this, a review of literature targeting social media-enabled PD has revealed a range of conclusions, benefits, challenges and finally gaps that can all be associated with its potential implementation into HE teachers’ PD plans. Despite the need to respect the views of sceptical teachers by not rushing the introduction of SNSs, the benefits of the practice outweigh the challenges. This will be explained in the subsequent discussion section, along with concluding remarks and future recommendations.

### General Conclusions

It is no secret that teachers’ overall dissatisfaction with formal, events-based PD has been steadily growing in recent years (Guskey, 2002; Oddone et al., 2019). While certain institutions and teachers maintain pertinent reasons for remaining loyal to this physical ‘one size fits all’ PD approach, namely due to the professional legitimacy attached to these events alongside their measurability, these reasons for impeding the advancement of PD are unlikely to hold for much longer. Aside from the semi-predictable catalysts of rapidly evolving technology and teachers’ needs to expand their PLNs (Trust et al., 2017) in response to growing competition, the WFH directive has acted as an unexpected accelerator in the shift towards this new form of social media-enabled PD. For tutors who now spend their working week in physical isolation facing their screens, personalised PD delivered across a variety of learning spaces, including SNSs, is no longer a desired luxury but rather, it is an urgent necessity. Stakeholders should not resist this shift towards a more flexible and blended version of PD (Postholm, 2012) that individuals can engage with according to their evolving needs (Trust et al., 2017). Perhaps now, when faculty find themselves in unfamiliar working circumstances, is an opportune time to implement this new approach.

Social media’s growing ubiquity, alongside technological breakthroughs in areas such as 5G and mobile learning devices, makes it a safe bet as a future PD arena. Despite this generally optimistic outlook, there is little evidence to suggest that educators and institutions should invest in any one particular SNS. Twitter may be the firm favourite for HE professionals today (O’Keeffe, 2016; Marin & Tur, 2014), but there is little to convince researchers as to specifically why it, and not similar competitors, holds this position. Add to this the lightning speed at which social media has evolved in the past decade (Kapoor et al., 2018), and one could be forgiven for recommending that teachers familiarize themselves with a plethora of platforms. They are unlikely to benefit from the mastery of a single platform for very long before a new one replaces it.

### Benefits

Among the many benefits of social media-enabled PD, the main appeal lies in its fluidity to adapt not only according to different teachers’ bespoke needs, but also according to a particular individual’s evolving requirements throughout varying stages of their career (Anders, 2018). The latter cannot be underestimated given the WFH policy’s radical impact on how many educators now operate. By integrating SNS PD, teachers and institutions are not only responding to the current WFH climate, in which HE professionals are still scrambling to function online, but they are also future-proofing themselves against pandemic-like calamities that could, once again, dramatically alter our working lives. The benefits of having social media-competent teachers the next time HE is thrown into turmoil through the sudden unavailability of physical learning spaces would be two-fold. Firstly, teachers would continue to engage in PD relatively uninterrupted and secondly, their added technological competence would better-equip them to work online at short notice.

Social media’s potential to engage educators in active, two-directional PD (Bedford, 2019) is another factor that should not be overlooked. In parallel to how twenty-first century teachers demand their learners actively engage in lessons, teachers themselves should follow suit by not settling for passive recipiency of PD material. Instead, tutors should take advantage of the interactional features of social media, in order to engage with like-minded professionals and leave their mark in the community by responding to online material. Not only is this likely to result in teachers who are more current with practice, it is also likely to combat some of the emotional well-being challenges (Anders, 2018; Carpenter & Krutka, 2014) associated with the WFH practice. Institutions should thus encourage educators to embrace social media to reap the benefits of NL, by actively engaging with the wider learning community and its resource repository (Goodyear et al., 2004).

### Challenges

From the challenges explored in the SLR, the lack of teacher ‘buy-in’ (Cater et al., 2013) encountered in some institutions is arguably the most significant obstacle (Marin & Tur, 2014). Social media’s relative infancy on the PD scene, coupled with its popularity as a recreational tool among younger generations (Lin et al., 2013), means that convincing all educators to adopt it in the short-term is unlikely. Given the contagious nature of negativity associated with new approaches (Ertmer & Ottenbreit-Leftwich, 2013), there is a danger that reluctance to accept SNS PD by a small number of teachers could spread across an institution and result in its collective delegitimization. Organizations should therefore reassure faculty by actively promoting the advantages of the underpinning notions of social media: NL and PLNs, prior to its implementation. By encouraging teachers to branch out to form new connections outside of their immediate physical PLNs (Forte et al., 2012), institutions can both reassure current social media-enabled PD practitioners of their independence from their close colleagues and also showcase the appeal of this practice to potential converts. The message sent to teachers should be that one’s PD commitment is no longer measured by a collection of attendance certificates, but rather by the growth and nurturing of their connections to like-minded professionals (Goodyear et al., 2004). Further research should be conducted into these benefits of social learning in the long-term, in order to more convincingly encourage teachers to invest in PD via social media.

Once the above-mentioned barrier of (il)legitimacy has been overcome, the other hurdles are comparatively easier to clear. The technical competence required to participate in social media-enabled PD (Donelan, 2016), for instance, is something that faculty have been tackling for some time (Ertmer & Ottenbreit-Leftwich, 2013). Teachers should, by now, be accustomed to being asked to develop their technological skills (Marin & Tur, 2014) and should no longer react to online training with the same levels of defensiveness reminiscent of the early digital era. A similarly clearable barrier is the vulnerability and fear that some teachers experience when participating in vast, online environments (Ranieri, 2019). In the long-term, educators can rest assured that SNS regulators are constantly improving their response to cyber-abuse, by more efficiently filtering hate-speech and deleting fake profiles, for instance. In the short-term, tutors can lower their exposure to the risks associated with online participation by anonymizing their personal information. This can be done through the use of pseudonyms and even avatars to substitute real pictures. Institutions should also consider implementing clear SNS-related policies that detail expectations of what faculty should and should not do on social media, in order to encourage staff to use these platforms without fear of reprisal from unintended breaches of policy. Future research should target these issues of technological up-skilling and safe online practice, in order to lower the entry barriers of social media-enabled PD for faculty.

### Gaps

Future research should target teachers’ qualitative in-depth perspectives of social media-enabled PD (Manca & Ranieri, 2017). This void in existing research could be tackled through phenomenography and case study approaches that closely track the day-to-day realities encountered by adopters of this young platform. By moving beyond the existing array of abstract descriptions of SNS PD (Trust el al., 2017), placing more individual case studies under the microscope will provide a more realistic picture of the practice. Shedding light on specific examples of how teachers engage in social media (O’Keeffe, 2016), be it through ‘commenting’ on videos, ‘sharing’ their own content or via any other form of engagement, will help stakeholders to overcome the challenges associated with the practice’s infancy (Luo et al., 2020), in order to legitimize it as a permissible platform for HE professionals to develop. Focusing on individual tutors’ experiences could also address current policy-related gaps, such as the additional personal costs incurred by faculty during the WFH period. Existing literature focuses extensively on figurative ‘costs,’ such as emotional strain and increased workload, but there is little information on the added physical costs that may include things like hardware replacement as personal computers slow down or the purchase of ergonomic office tools as home equipment becomes uncomfortable for extended use. Future research on this area would help to fuel debate as to how these costs should be covered in future WFH events.

Future studies should also address the gap regarding the partial integration of social media-enabled PD into institutions’ development plans. Instead of promoting polarization between different approaches to PD (Greenhalgh & Koehler, 2016), namely F2F workshop events versus the use of SNSs, future studies should focus on hybrid forms of PD that combine both approaches (Mirriahi et al., 2015). By adopting a blended PD plan (Evans et al., 2020), educators can aspire to ‘cherry-pick’ the most favourable elements from both approaches, whilst sidestepping the hurdles. A teacher may, for instance, engage in general F2F workshops that partly meet their needs, but then use a workshop’s content as a springboard to expand their PLN once the session has ended (Anders, 2018; Forte et al., 2012). This could be done by engaging online with participants with PD interests more specifically aligned to the teacher’s own goals after the workshop has ended, for instance, or by sharing material and reflections from the F2F workshop online via Twitter. A combination such as this is likely to satisfy an institution’s record-keeping requirements, since the tutor’s attendance would be logged, but at the same time encourage the educator not to settle for what is geographically and temporally available based on their physical location.

## Final Thoughts

This paper has argued that social media-enabled PD can and should be integrated into the PD plans of twenty-first century HE educators. The benefit of a PD experience that is bespoke to each individual cannot be overlooked, especially in the challenging WFH times we face today where many of us are physically isolated and in desperate need of tailored on-the-job training. The challenges experienced in some institutions related to teachers’ perceptions of the SNS platform can be tackled through the adoption of a hybrid model of PD, whereby traditional F2F events can be used to complement social media interaction. The emphasis should be on how SNSs can be used to extend each educator’s PLN; rather than on the SNS itself. Institutions should present social media-enabled PD as an extension of F2F PD; as opposed to its outright replacement. The balance of events-based workshops alongside social media-enabled PD will have to be decided on a case-by-case basis according to each institutions’ collective faculty attitude: universities facing significant resistance to the adoption of social media for instance should begin with a SNS ‘lite’ approach, until staff have acclimatized. PD programmes completely absent of social media, however, are unlikely to have a place in the future of HE.

## Data Availability

All relevant data, material and coding is included in the manuscript.

## Appendix 1—PRISMA Checklist

From: Moher D, Liberati A, Tetzlaff J, Altman DG, The PRISMA Group (2009). Preferred Reporting Items for Systematic Reviews and Meta-Analyses: The PRISMA Statement. PLoS Med 6(6): e1000097. https://doi.org/10.1371/journal.pmed1000097

## Appendix 2- Literature Matrix Sample

Adapted from:

Walden University (2020, September 13) Common Assignments: Literature Review Matrix. https://academicguides.waldenu.edu/writingcenter/assignments/literaturereview/matrix

## Appendix 3- Screening Document

Self-made 14/10/2020.

## Appendix 4- Synthesis Notes Document

Self-made 14/10/2020.

## Appendix 5- De Laat’s Networked Learning Structure Concept Map Example

From: De Laat, M. (2012). Enabling professional development networks: How connected are you?: Open Universteit.

## Appendix 6- Cook et al.’s Online PLN Criteria

From: Cook, R. J., Jones-Bromenshenkel, M., Huisinga, S., & Mullins, F. (2017). Online Professional Learning Networks: A Viable Solution to the Professional Development Dilemma. Journal of Special Education Technology, 32(2), 109–118. https://doi.org/10.1177/0162643417696930
